# Evaluation of Different Biomarkers to Predict Individual Radiosensitivity in an Inter-Laboratory Comparison–Lessons for Future Studies

**DOI:** 10.1371/journal.pone.0047185

**Published:** 2012-10-23

**Authors:** Burkhard Greve, Tobias Bölling, Susanne Amler, Ute Rössler, Maria Gomolka, Claudia Mayer, Odilia Popanda, Kristin Dreffke, Astrid Rickinger, Eberhard Fritz, Friederike Eckardt-Schupp, Christina Sauerland, Herbert Braselmann, Wiebke Sauter, Thomas Illig, Dorothea Riesenbeck, Stefan Könemann, Normann Willich, Simone Mörtl, Hans Theodor Eich, Peter Schmezer

**Affiliations:** 1 Department of Radiotherapy, University Hospital of Muenster, Muenster, Germany; 2 Leibniz Institute for Age Research – Fritz Lipmann Institute (FLI), Jena, Germany; 3 Institute of Radiation Biology, Helmholtz Centre Munich – German Research Centre for Environmental Health, Neuherberg, Germany; 4 Institute of Biostatistics and Clinical Research, University of Muenster, Muenster, Germany; 5 Institute of Molecular Radiation Biology, Helmholtz Centre Munich – German Research Centre for Environmental Health, Neuherberg, Germany; 6 Institute of Epidemiology, Helmholtz Centre Munich – German Research Centre for Environmental Health, Neuherberg, Germany; 7 Division of Epigenomics and Cancer Risk Factors, German Cancer Research Centre (DKFZ), Heidelberg, Germany; 8 Federal Office for Radiation Protection, Department of Radiation Protection and Health, Oberschleissheim, Germany; National Taiwan University, Taiwan

## Abstract

Radiotherapy is a powerful cure for several types of solid tumours, but its application is often limited because of severe side effects in individual patients. With the aim to find biomarkers capable of predicting normal tissue side reactions we analysed the radiation responses of cells from individual head and neck tumour and breast cancer patients of different clinical radiosensitivity in a multicentric study. Multiple parameters of cellular radiosensitivity were analysed in coded samples of peripheral blood lymphocytes (PBLs) and derived lymphoblastoid cell lines (LCLs) from 15 clinical radio-hypersensitive tumour patients and compared to age- and sex-matched non-radiosensitive patient controls and 15 lymphoblastoid cell lines from age- and sex- matched healthy controls of the KORA study. Experimental parameters included ionizing radiation (IR)-induced cell death (AnnexinV), induction and repair of DNA strand breaks (Comet assay), induction of yH2AX foci (as a result of DNA double strand breaks), and whole genome expression analyses. Considerable inter-individual differences in IR-induced DNA strand breaks and their repair and/or cell death could be detected in primary and immortalised cells with the applied assays. The group of clinically radiosensitive patients was not unequivocally distinguishable from normal responding patients nor were individual overreacting patients in the test system unambiguously identified by two different laboratories. Thus, the *in vitro* test systems investigated here seem not to be appropriate for a general prediction of clinical reactions during or after radiotherapy due to the experimental variability compared to the small effect of radiation sensitivity. Genome-wide expression analysis however revealed a set of 67 marker genes which were differentially induced 6 h after in vitro-irradiation in lymphocytes from radio-hypersensitive and non-radiosensitive patients. These results warrant future validation in larger cohorts in order to determine parameters potentially predictive for clinical radiosensitivity.

## Introduction

About 5–10% of the patients treated with radiotherapy show particularly early and/or severe side reactions of the co-irradiated normal tissue without any indication for predispositions like previous diseases or exogenous factors [1]. The underlying causes for such (hyper)radiosensitivity remain obscure and cannot be reliably predicted due to the lack of appropriate biomarkers.

Pre-therapeutic identification of radiosensitive patients would allow improvement of individual patients' treatment; e.g., interruptions during radiotherapy with the known negative consequences for tumour control [2] could be avoided by early therapeutic intervention or by dose reduction. Patients with increased radiosensitivity could be excluded from dose intensification studies and could be informed about their increased risk to decide about different therapy options. Conversely, non-radiosensitive patients without risk factors might profit from dose escalation [3].

Several projects aiming at cellular and molecular mechanisms and biomarkers of individual radiosensitivity have been reported [4]. The results were conflicting.

Comparability of such single studies is hampered due to many factors including i) the size and the clinical heterogeneity of the patient collectives, ii) the study designs (e.g. retrospective versus prospective) [5] iii) the poor correlation of the biological endpoints used, and iv) differences in clinical characterisation of hypersensitive patients based on IR-related side reactions [6]. Apart from the experimental design, the question arises whether distribution of radiosensitivity in the population follows a Gaussian distribution or if hypersensitive patients form a separate peak apart from the non-radiosensitive individuals [7].

We here designed a multicentric, multi-parametric, blind, age- and sex-matched case-control study approach to experimentally address clinical radiosensitivity. Five different laboratories investigated in parallel different radiobiological endpoints in identical aliquots of encoded primary peripheral blood lymphocytes (PBLs) and derived EBV-transformed lymphoblastoid cell lines (LCLs) of 15 matched sample pairs from clinically radiosensitive vs. normal responding tumour patients and 15 lymphoblastoid cell lines from age- and sex-matched healthy controls of the KORA study.

## Materials and Methods

### Ethics Statement

The study was approved by the Ethics Committee of the University of Münster. All subjects gave their written informed consent.

### Study design

Five different laboratories participated in this study. Three laboratories [Münster (A), München (B) and Jena (C)] validated the Annexin V/Propidiumiodide (PI)-based cell death assay. Two laboratories [Heidelberg (D) and BfS, München (E)] investigated DNA damage induction and repair using the alkaline Comet assay and another two laboratories [München (B) and Jena (C)] investigated DNA damage-induced phosphorylation of the histone H2AX using the γH2AX assay. In addition gene expression profiles were determined in one laboratory [Heidelberg (D)]. Patient recruitment, blood collection and lymphocyte preparation were carried out at the Department of Radiotherapy of the University Hospital of Münster (A).

Cellular assays were performed in paired series of matched samples in a blind manner in each participating laboratory. Samples were decoded only after experimental analysis and data evaluation.

### Patient recruitment

Within the scope of a former project dealing with individual radiosensitivity more than 550 patients attending the Department of Radiotherapy due to head and neck or breast cancer were characterised in detail regarding their acute toxicity during radiotherapy [8]. The clinical radiosensitivity was evaluated by classifying the reaction of the skin in the radiation field and in case of head and neck tumours additionally the reaction of the mucosa in mouth and pharynx. Qualitative and quantitative classification of clinical reactions to skin, mucosa and most other organs at risk was performed according to the RTOG/European Organisation for Research and Treatment of Cancer (EORTC) criteria published by Perez and Brady 1993 [9]. Only for lymphedema, nausea/vomiting and nutrition the Common Toxicity Criteria (CTC) score was used (National Cancer Institute, USA, 1988, Version 1.0 [10]), but these endpoints were not used for definition of clinical radiosensitivity. Both classifications were utilized using the German translation published 1998 by Seegenschmiedt [11]. Classification was done independently by three experienced and particularly trained physicians of the radiotherapy department. Basic data of the involved patients and possible factors which may influence the side reactions and experimental investigations, like comorbidity and use of medicines, tumour characterisation, pretreatment, tumour treatment so far, radiotherapy as well as general condition, smoking habit, consumption of alcohol and mental stability were documented [8]. Patients were defined as radiosensitive if they fulfilled one of the following criteria: Head and neck cancer patients: acute grade 1 reaction below 14 Gy cumulative dose, acute grade 2 reaction below 30 Gy or acute grade 3 reaction more than three weeks after therapy pause/end of therapy. Breast cancer patients: acute grade 1 reaction below 10 Gy, acute grade 2 reaction below 20 Gy or acute grade 3 reaction on the breast. Using these criteria, 15 patients (10 breast carcinomas, 5 head and neck carcinomas) displaying acute clinical radiation hypersensitivity were available at the beginning of this study without a tumour recurrence or secondary malignancy. Fifteen non-radiosensitive patients identified from the same population were included as age- and sex-matched controls (Table 1 and 2) and 15 lymphoblastoid cell lines from age- and sex-matched healthy controls of the KORA study (http://www.helmholtz-muenchen.de/kora). Blood samples (250 ml each) for this study were collected from these 30 patients two to five years following radiotherapy, including an evaluation of late reactions. Sample preparation and management as well as EBV-transformation were described previously [12].

**Table 1:** Late toxicity grade in acutely radiosensitive (s) and non-radiosensitive (ns) patients suffering from breast cancer. Each patient is indicated with an identification number (ID).

ID	group	skin- teleangiectasia	skin- pigmentation	skin - ulceration	skin - atrophia	skin fibrosis	lymphedema breast	objective clinical outcome	subjective clinical outcome
1a	ns	0	0	0	0	0	0	1	1
1b	s	1	0	0	1	2	1	2	2
2a	ns	0	0	0	0	1	0	1	1
2b	s	1	0	0	1	1	1	2	2
3a	s	0	0	0	0	1	0	2	2
3b	ns	0	0	0	1	1	0	2	2
4a	ns	0	0	0	0	0	0	1	1
4b	s	2	0	0	2	1	1	2	2
5a	ns	0	0	0	0	0	0	1	1
5b	s	0	0	0	1	2	2	2	2
6a	s	0	0	0	1	1	1	2	2
6b	ns	0	0	0	0	0	0	1	1
7a	s	1	0	0	2	1	0	2	2
7b	ns	0	0	0	0	0	0	1	1
8a	s	2	1	0	2	2	0	3	3
8b	ns	0	0	0	0	0	0	2	2
10a	s	0	0	0	1	1	0	1	1
10b	ns	0	0	0	0	0	0	2	2
14a	s	3	1	0	2	2	2	3	3
14b	ns	0	0	0	0	0	0	1	1

Matched pairs with lower late toxicity of the acute radiosensitive patients are indicated in yellow.

**Table 2:** Late toxicity grade in acutely radiosensitive (s) and non-radiosensitive (ns) patients suffering from head and neck cancer. Each patient is indicated with an identification number (ID).

ID	group	skin- teleangiectasia	skin- pigmentation	skin - ulceration	skin - atrophia	skin - fibrosis	mucosa	xerostomy	paryngeal score	nutrition score	lymphedema head
9a	ns	1	0	1	1	1	1	2	0	2	0
9b	s	0	0	0	2	1	2	1	1	0	1
11a	s	1	1	0	1	1	1	2	0	0	2
11b	ns	0	0	0	0	1	2	2	0	0	1
12a	ns	1	0	0	1	1	1	1	0	0	1
12b	s	1	1	0	1	1	1	2	1	1	2
13a	ns	0	1	0	1	1	1	1	0	1	2
13b	s	3	2	0	2	2	2	2	3	2	1
15a	s	1	0	0	0	0	3	2	1	2	0
15b	ns	1	0	0	1	1	0	1	0	0	1

Matched pairs with lower late toxicity of the acute radiosensitive patients are indicated in yellow.

### Isolation of lymphocytes, transport and delivery conditions for the biological material

240 ml peripheral blood was collected under the addition of heparin (5,000 I.E. per 50 ml) and PBLs were isolated immediately by density gradient centrifugation as previously described [13]. PBLs were aliquoted in 10×10^6^ cells per ml and stored in liquid nitrogen prior to delivering them to the cooperating laboratories. Additional 10 ml blood was collected under the addition of EDTA (1.6 mg per ml) and was directly used for preparing the LCLs. Patient-derived PBLs were sent as coded samples on dry ice to the participating laboratories (A to E). To control for potential transport-related cellular stress, the sending laboratory (A) also stored its samples on dry ice during the time of the transport.

### Cultivation of the cells

EBV transformed lymphocytes were cultivated according to a standardised protocol which was performed in all participating groups. Cells were grown in RPMI 1640 plus L- glutamine (PAA-laboratories Nr. E15885), 1% penicillin/streptomycin (Gibco Nr. 15140–122) and 20% heat inactivated fetal calf serum (PAA-Laboratories Nr. A15-104) and cultured at 37°C and 5% CO_2_. Cell density did not exceed 1×10^6^ cells/ml. At least 10 aliquots of the second or third passage were gently cryo conserved in medium supplemented with 10% DMSO and stored in liquid nitrogen (density of 2×10^6^ cells) and used for all following experimental investigations.

### Cell death (Annexin V/PI)

Following irradiation cell death was measured using the Annexin V/PI assay (Invitrogen, Karlsruhe, Germany). All test- and measuring parameters were standardised between the participating laboratories; in detail the whole procedure has been described recently [12].

### Alkaline single-cell gel electrophoresis assay (Comet assay)

Primary lymphocytes were grown in medium as described above overnight. EBV transformed cell lines (LCLs) were cultivated for 10 days prior to irradiation. Matched pairs of primary cells, LCLs of patients and matched healthy control cell lines (KORA cohort) as well as two internal standards (primary lymphocytes and one healthy patients cell line) were investigated always in one experimental run. Irradiation of cells was performed differently in the two laboratories. They were either embedded in agarose on microscopical slides or collected in reaction tubes on ice and irradiated with 5 Gy using a ^137^Cs radiation source with a dose rate of 0.54 and 0.575 Gy/min, respectively. Unirradiated controls were analysed in parallel. DNA repair was assessed by incubating the samples for 15 and 60 minutes after irradiation at 37°C. For the Comet assay analysis, the cellular genomic DNA was electrophoresed under alkaline conditions, according to the protocol described in Rosenberger et al. 2011 [14] with modification of the second layer which was 0.7% low melting agarose (Seakem) and electrophoresis was performed at 4°C. DNA damage was assessed by the parameters “Olive Tail Moment” (OTM) and “DNA intensity in tail” (Tail DNA in %). Analysis and evaluation of cellular damage was performed by fluorescence microscopy using a fully automated cell scanning system Metafer-4 (Metasystems, Altlußheim, Germany) which is described in Schunck et al. 2004 [15]. The controls were used to normalise the values of the matched sample pairs to eliminate experimental variations.

### DNA damage-induced phosphorylation of H2AX (γH2AX)

PBLs and LCLs of the 15 matched sample pairs and matched healthy control cell lines were used to investigate DNA damage-induced phosphorylation of the histone H2AX variant to γH2AX at 15 minutes, 1 h, 4 h and 24 h after irradiation with 2 Gy gamma rays. Upon IR, cells were harvested and fixed, first in 1.5% Formaldehyde and afterwards in ice cold 70% ethanol. The samples were collected at −20°C, again permeabilised in 0.25% Triton X-100, blocked by 5% Goat serum and labelled for their H2AX phosphorylation using the monoclonal Anti-phospho-Histone H2A.X (Ser139) antibody, clone JBW301 (UPSTATE, Lake Placid, USA). Fluorescence labelling took place by using an Alexa Fluor 488 conjugated goat anti-mouse antibody (Invitrogen, Karlsruhe, Germany) and fluorescence intensity was measured on a flow cytometer [16] (München: BD LSR II, Becton Dickinson Biosciences, Heidelberg, Germany; Jena: FACS Calibur, Becton Dickinson Biosciences, Heidelberg, Germany). For statistical analysis the median of fluorescence at 530 nm (530/30 BP) was used to calculate the relative H2AX phosphorylation.

### Statistics

A total of 30 eligible patients who underwent blood tests at the Department of Radiotherapy of the University Hospital of Münster were included in the analysis. In addition to the acquisition of the basic clinical data, laboratory data were evaluated in all participating centres. For the inter-laboratory comparisons Spearman's rank correlations and Bland-Altman analyses were performed, to determine the degree of agreement between the measurements within one laboratory experiment in different centres. The results of the measurements were analysed by nonparametric ANOVA for repeated measures (Friedman test) and pairwise Wilcoxon signed rank tests, respectively.

Dose-response relationship between the dose and the severity of their effect was determined in an analogous manner. The Mann-Whitney U test was performed to compare for statistically significant differences between the groups of radiosensitive and non-radiosensitive patients.

Inductive statistical analyses were performed to account for clusters of correlated observations of individual subjects. Linear models of the target parameters were established and fitted by generalised estimating equations (GEE), applying an exchangeable working correlation within clusters.

Besides the analyses mentioned above, a transformed matrix with all measured parameters was constructed, to test for different cumulative frequencies in conspicuous samples between radiosensitive and non-radiosensitive patients.

## Results

### Correlation of late clinical reactions with acute toxicity

The classification of the late reactions, performed at the time of blood donation, showed a good correlation (80%) to the radiosensitivity in view of acute toxicity. In breast cancer patients, eight of ten patients previously described as acutely radiosensitive showed both increased numbers and severity of late side reactions as compared to the matched patients without acute radiosensitivity. In patients suffering from head and neck cancer, this was seen in four out of five pairs (see Table 1 and 2).

### Apoptosis (Annexin V/PI)

The three participating laboratories observed a highly significant dose-dependent increase of IR-induced apoptosis in the individual samples to a very similar extent. However, comparing the mean values of Annexin V-positive and Annexin V/PI-double-positive cells derived from peripheral blood lymphocytes (PBLs) of the radiosensitive cohort with the mean values of the non-radiosensitive cohort did not reveal a significant difference. In addition, the few individual outliers detected in different labs were not confirmed in other labs.

As has been reported previously, the Annexin V/PI assay failed to detect Annexin V-positive and Annexin V/PI-double-positive cells in immortalised lymphoblastoid cell lines (LCL) upon irradiation with low doses of IR [12]. The differences in IR-induced apoptosis found in primary cells of different individuals were thus not detectable in the corresponding LCL derivatives and all statistic evaluations are based on PBL results.

In the interlaboratory analysis, the reliability of the Annexin V/PI-based apoptosis assay was tested. Two laboratories revealed low rates of Annexin V-positive cells (mean centre B 6.7% and mean centre C 7.8%) and high rates of Annexin V/PI-double-positive cells (mean centre B 72.4% and centre C 65.9%) while one laboratory (centre A) revealed higher rates of Annexin V-positive (mean 22.1%) and lower rates of Annexin V/PI-double-positive cells (mean 22.9%). The interlaboratory comparision by Bland Altman analysis revealed large systematic differences between results of centre A and centre B (bias +18.8% for Annexin V-positive and −53.3% for Annexin V/PI-double-positive) and of A and C (bias +17.1% for Annexin V-positive and −48.7% for Annexin v/PI-double-positive). Results of centre B and C showed better correlation (bias −1.7% for Annexin V-positive and +4.5% for Annexin V/PI-double-positive).

However, within 15 matched pairs, no sample was unambiguously and independently identified by all three participating laboratories to demonstrate *in vitro* hypersensitivity that matched the clinical hypersensitivity (Table 3).

**Table 3:** Comparison between non-radiosensitive vs. radiosensitive patients for apoptosis and necrosis in each laboratory within the different dose rates.

Apoptosis/Necrosis	Non-radiosensitive vs. radiosensitive		
Dose (Gy)	0.0	0.4	0.8
Centre A	0.624	0.512	0.305
Centre B	0.830	0.645	0.798
Centre C	0.389	0.395	0.373

P values were determined by Mann-Whitney U test. The differences were statistically not relevant as indicated by the high p values >0.05.

### DNA damage induction and repair (Comet assay)

The induction and repair of DNA lesions such as DNA strand breaks are key factors that modulate individual radiation sensitivity and may thus be altered in individuals showing severe side effects of the co-irradiated normal tissue. The induction and repair of radiation-induced DNA breakage was evaluated in the patient-derived cells by two laboratories (D and E) using the alkaline Comet assay to investigate its predictive value.

**Figure 1:**
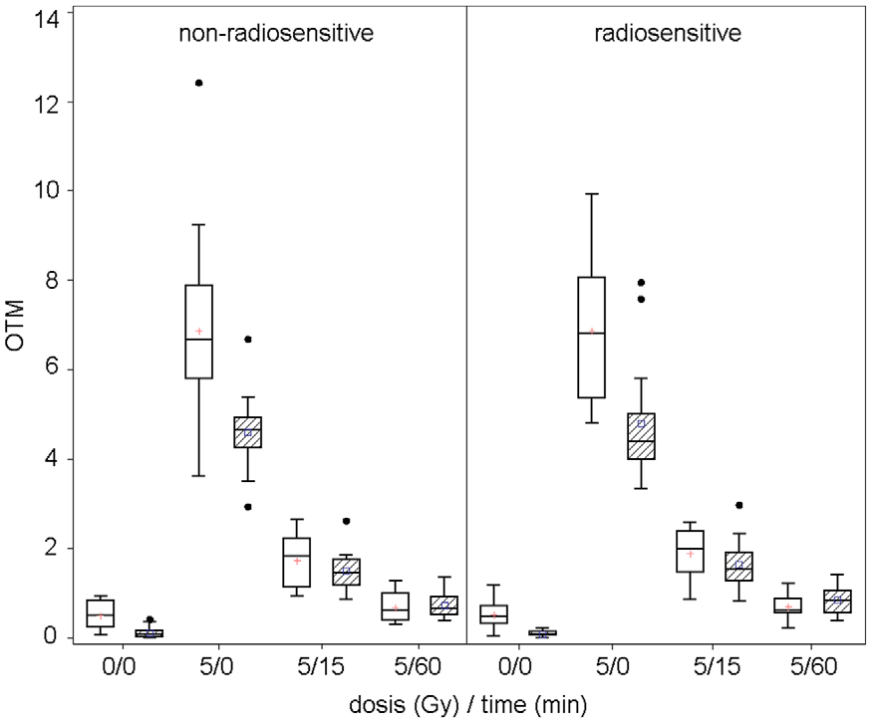
Dose-response relationship of Olive tail moment (OTM) in PBLs at different time points. Data from two different centres E (white boxes) and D (hatched boxes) are presented. Comet assay of both laboratories revealed similar results by investigating OTM in PBLs directly after irradiation with 5 Gy and at different time points. Non-radiosensitive and radiosensitive individuals were not distinguishable.

Both laboratories were able to show a significant dose-response relationship of Olive tail moment (OTM) (Figure 1) and %Tail DNA (data not shown) for all investigated samples.

The absolute values were in good agreement for non-irradiated and repaired samples but differed significantly between the two laboratories for the irradiated probes. The coefficient of variation for the standard control was similar in both laboratories. Strong radiation-induced DNA breakage could be detected in both laboratories. Although the LCLs were derived from the PBLs, LCLs and PBLs were different in their repair capacities. After 15 minutes, LCLs repaired 83% of the damage while PBLs showed only 60% repair capacity. None of the laboratories could identify a significant difference in the reaction of the clinically radiosensitive as compared to the non-radiosensitive patients. Outliers have been identified in both laboratories but the identified individuals were not in agreement between the two laboratories (Table 4).

**Table 4:** Spearman rank correlation coefficient between the 2 laboratories D and E for DNA damage, assessed by the target parameters OTM and % Tail DNA standardised.

PBL- Olive Tail Moment Gy/min	Spearman-Corr. (N = 30)	95%-confidence interval	p-value
0/0	0.149	[−0.225270; 0.481695]	0.434
5/0	−0.012	[−0.370311; 0.350143]	0.951
5/15	0.114	[−0.258947; 0.453749]	0.553
5/60	0.347	[−0.021336; 0.624928]	0.060

PBL –% Tail DNA Gy/min	Spearman-Corr. (N = 30)	95%-Confidence Interval	p-value
0/0	0.149	[−0.225908; 0.481179]	0.436
5/0	0.095	[−0.276596; 0.438521]	0.622
5/15	0.150	[−0.225004; 0.481911]	0.433
5/60	0.455*	[0.105491; 0.696400]	0.011(*)

For the standardised comparison of both centres, all raw data were transformed by multiplicative standardisation, P-values were calculated to assess whether the association between both laboratories is significantly different from zero with a p-value<0.05 (*). The association was only statistically significant for 5Gy 60 min within the PBLs for Tail DNA. The positive correlation coefficient with a positive 95%-confidence interval indicates that the data of centre E tends to increase when the data of centre D increase.

(Abbreviations: PBL: Peripheral blood lymphocytes).

### Induction of DNA double-strand breaks by histone H2AX phosphorylation (γH2AX)

**Figure 2:**
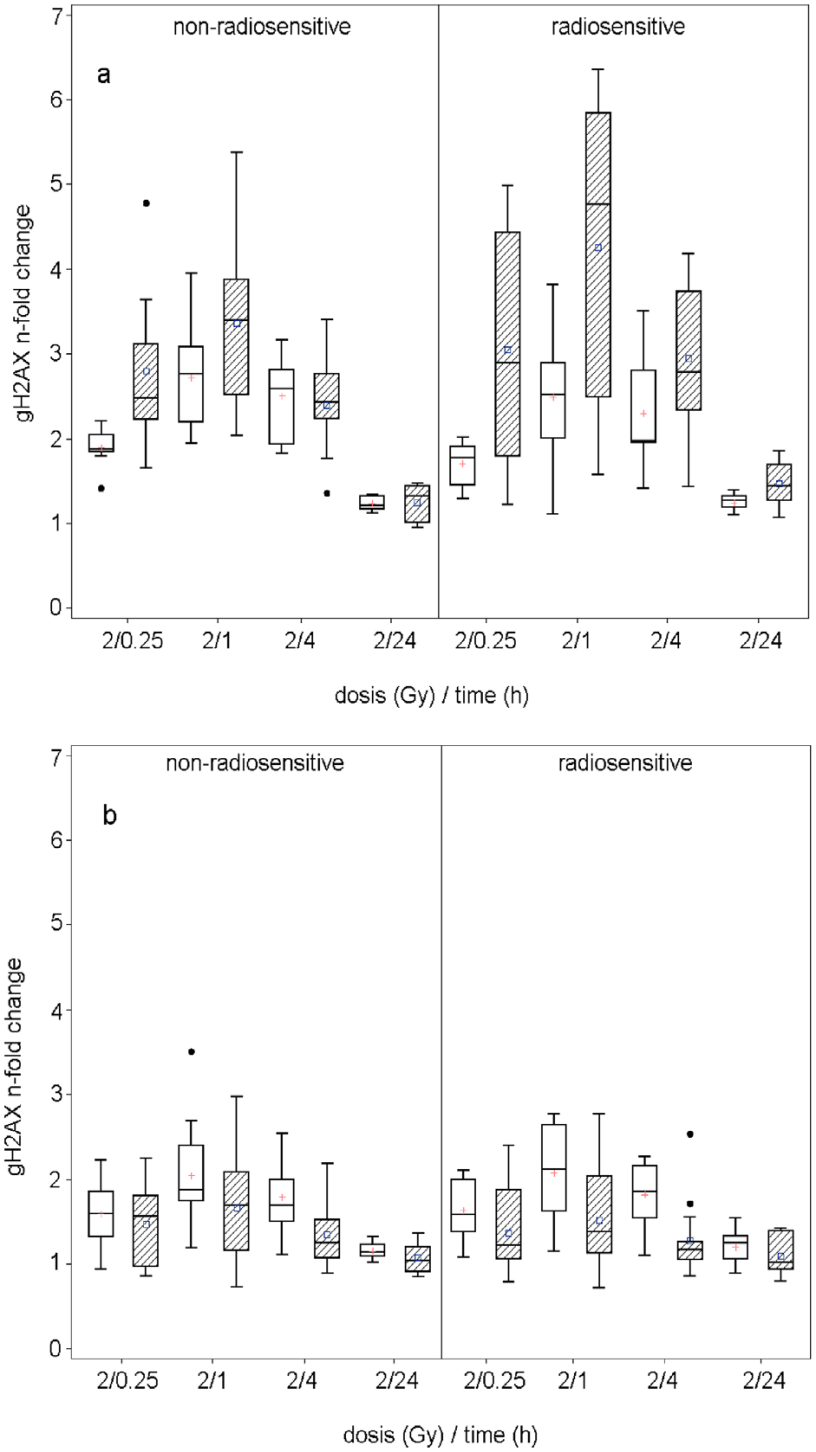
Dose-response relationship of Gamma-H2AX at different time points from two different centres (white boxes – centre C, hatched boxes – centre B). Each measured value corresponds to the n-fold change to the time point at 0 Gy after 24 hours. (a) Data for the PBLs: Box plots only include samples with an initial cell viability of higher than 80%. (b) Data for LCLs over all samples.

Both involved laboratories (B and C) found an induction of histone H2AX phosphorylation after irradiation in PBLs (Figure 2 a). Maximum H2AX phosphorylation was detected one hour after irradiation and DNA repair kinetics can be followed by decreasing phospho-H2AX levels. Twenty four hours after irradiation phosphorylation of H2AX is reduced close to the level of non-irradiated cells, indicating complete repair. Remarkably, both laboratories detected a great variability in H2AX phosphorylation at the early time points after irradiation, indicating variation in DNA repair kinetics, while after 24 h all samples displayed rather homogenous levels of phosphorylated H2AX close to the background levels. Comparison of phospho-H2AX levels at the individual time points showed no significant differences between PBLs of radiosensitive and non-radiosensitive patients.

The investigation of the corresponding EBV-immortalised LCLs showed less overall IR-induced H2AX phosphorylation than the corresponding PBLs in both laboratories (Figure 2 b). However, in agreement to the findings in PBLs, LCLs displayed a highly variable induction of H2AX shortly after irradiation followed by a rather complete repair at 24 h in all samples.

The comparison of the cellular γH2AX-fluorescence levels with clinical radiosensitivity of the respective patients showed no clear phenotypic correlation, neither for the PBLs nor for the LCLs in either laboratory. However, a higher variation of the γH2AX induction values in the PBLs of the radiosensitive cohort compared to the non-radiosensitive one was found in laboratory B. Taken together, measurement of radiation-induced histone H2AX phosphorylation at the indicated time points and doses was not able to identify samples from patients displaying clinical radiosensitivity.

### Comparison between tumour entities

Head and neck cancer patients (n = 10) revealed significant higher apoptosis (p<0.0001) and γH2AX values on average (p = 0.0008) in PBLs compared to the breast cancer patients (n = 20), while the Comet assay data showed a significantly lower OTM (p = 0.0126) and % Tail DNA (p = 0.0205) (Table 5).

**Table 5:** Results of the comparison between head and neck tumour (HN-Ca) and breast cancer patients (Ma-Ca) within a generalised estimating equations (GEE) analysis, adjusted for laboratory, irradiation dose and sensitivity group.

Assay	Parameter	Reference Ma-Ca	Estimator HN-Ca	Standard Error	95% Confidence limits	p-value
Apoptosis	Apoptosis (%)	0.000	6.3670	1.4911	[3.444;9.290]	<0.0001*
	Necrosis (%)	0.000	−4.8982	3.1442	[−11.061;1.264]	0.1193
γH2AX	PBLs spontaneous	0.000	394.4866	117.89	[163.408;625.565]	0.0008*
	LCLs spontaneous	0.000	192.4205	157.179	[−115.645;500.486]	0.2209
Comet assay	PBLs – OTM non-standardised	0.000	−0.3361	0.1347	[−0.6002; −0.072]	0.0126*
	PBLs – %Tail DNA non-standardised	0.000	−1.6470	0.7111	[−3.0407; −0.2533]	0.0205*
	
	LCLs – OTM non-standardised	0.000	−0.0895	0.1447	[−0.3731;0.1940]	0.5359
	LCLs – %Tail DNA non-standardised	0.000	0.0394	0.7473	[−1.4252;1.5041]	0.9579
Comet assay	PBLs – OTM standardised	0.000	−0.5011	0.2152	[−0.9229; −0.0793]	0.0199*
	PBLs – %Tail DNA standardised	0.000	0.3216	2.8845	[−5.3319;5.9751]	0.9112
	
	LCLs – OTM standardised	0.000	0.0711	0.2668	[−0.4518;0.5939]	0.7899
	LCLs – %Tail DNA standardised	0.000	8.9701	7.1309	[−5.0062; 22.9463]	0.2084

Columns represent the assays, the target parameter names, the estimated parameter value, the standard error of the parameter estimate, the confidence intervals and the associated p-value for testing the significance of the parameter to the model. P-values less than 0.05 are considered significant (*). The estimator is a parameter that indicates the average value, on which the head and neck cancer patients have less or greater values compared to breast cancer patients.

**Table 6:** Required number of samples per group (radiosensitive and non-radiosensitive, respectively) to detect a significant difference between both groups (power  = 80%, significance level  = 5%) for a given standard deviation and effect size.

Parameter	Cells	SD	Effect size	Delta	Sample size per group
OTM	PBL	0.3	0.25	0.075	252
		0.3	0.5	0.15	64
		0.3	1	0.30	17
		0.3	2	0.60	5
		0.6	0.25	0.15	252
		0.6	0.5	0.30	64
		0.6	1	0.60	17
		0.6	2	1.20	5
	LCL	0.2	0.25	0.05	252
		0.2	0.5	0.10	64
		0.2	1	0.20	17
		0.2	2	0.40	5
		1.0	0.25	0.25	252
		1.0	0.5	0.50	64
		1.0	1	1.00	17
		1.0	2	2.00	5

Parameter	Cells	SD	Effect size	Delta	Sample size per group
% Tail DNA	PBL	2.0	0.25	0.50	252
		2.0	0.5	1.00	64
		2.0	1	2.00	17
		2.0	2	4.00	5
		4.5	0.25	1.125	252
		4.5	0.5	2.25	64
		4.5	1	4.50	17
		4.5	2	9.00	5
	LCL	1.5	0.25	0.375	252
		1.5	0.5	0.75	64
		1.5	1	1.50	17
		1.5	2	3.00	5
		5.3	0.25	1.325	252
		5.3	0.5	2.65	64
		5.3	1	5.30	17
		5.3	2	10.60	5

The calculations were reduced by the 2 endpoints of repairing after 15 and 60 minutes of 5Gy irradiation, since these points of time appear to need the lowest sample sizes. With regard to the standard deviation the minimal and maximal values from the original dataset for the two timeframes were selected.

## Discussion

This study was initiated to validate cellular parameters derived from patient cells for their capacity to predict the radiosensitivity of patients' co-irradiated normal tissue. The parameters were examined in PBLs and corresponding LCLs. This allowed also to test whether LCLs may serve as surrogates for the fragile PBLs.

More than 550 patients undergoing radiotherapy due to head and neck or breast cancer at the Department of Radiotherapy in Münster, Germany, were thoroughly characterised regarding their acute toxicity. Thirty of these patients were detected to fulfil the criteria of radiosensitive patients, and these patients were asked for a blood donation two to five years after radiotherapy. 15 patients could be matched with non-radiosensitive patients of the same collective and were thus included as 15 matched pairs for the study described here. The other 15 radiosensitive patients had to be excluded due to tumour recurrence or refusal of informed consent. Comparison of the initially documented acute side effects, which led to the characterisation of clinical radiosensitivity, clearly correlated in 80% of the matched pairs with both increased numbers and severity of late sequelae seen two to five years after radiotherapy. However, in 20% (2 pairs with breast cancer and 1 pair with head and neck cancer) no strong difference in the development of late sequelae between patients initially categorized as radiosensitive vs. non-radiosensitive could be found, these matched pairs are highlighted in table 1 and 2. Based on this data, we conclude that our radiosensitive cohort is well distinguished from the non-radiosensitive cohort. The strong correlation between acute clinical radiosensitivity and late side effects supports our presumption of a genetic contribution to individual radiosensitivity.

According to our multicentric study design, identical lymphocyte aliquots were distributed as coded samples to the five partners that performed highly standardised multi-parametric tests in parallel. Furthermore, to minimise the risk of a selection bias, we picked out an eligible non-radiosensitive age- and sex-matched control cohort. Statistical comparison of test results thus enabled us to prove the reproducibility of the measurements of specific parameters and their usefulness as potential indicators for biomarkers of radiosensitivity.

IR-induced apoptosis in PBLs was successfully investigated in three different laboratories by using a highly standardised Annexin V/PI protocol. However, apoptosis of PBLs was found to be unsuitable to unequivocally predict the individual clinical radiosensitivity of cancer patients. A clear dose response relationship was found in all cases but radiosensitive individuals were not to be distinguishable from their non-radiosensitive matched controls. Thus, apoptosis measured with Annexin V does not correlate with individual radiosensitivity in our multicentric study. Also other research groups [17], [18], using different cell death measuring methods, could not confirm the studies of Crompton et al. [19] who showed a reduced IR-induced cell death in radiosensitive cancer patients by using the subG1 assay [19], [20]. On the other hand, a recently published study on head and neck cancer supports the association of low apoptosis values in patients PBLs and radiation induced severe xerostomia in normal tissue [21]. Also late radiation-induced toxicity was described to be predictable by low apoptosis values in CD4 and CD8 lymphocytes [22]. Thus, results are quite different and may depend on study design, sample treatment and measuring methods.

Furthermore, using the parameters tested here LCLs do not reflect the physiological properties of the corresponding PBLs with regard to IR-induced apoptosis measured with the Annexin V test. Their value to predict clinical radiosensitivity is thus highly questionable [12]. This notion is strongly supported by the gene expression data. Compared to the PBLs, the corresponding LCLs showed a completely different gene expression profile and especially apoptosis inhibiting genes did not show up [23]. Other research groups also found highly proliferating LCLs to express EBV-specific proteins with anti-apoptotic activity like BamH1 rightward reading frame (BHRF1) [24], [25], [26], [27]. Even in the unirradiated LCLs, about 4,800 genes from more than 170 different pathways were significantly altered as compared to PBLs which results in far reaching alterations in the transcriptome of the cells with consequences on all considered endpoints. Irradiation experiments revealed nearly no further change in the gene expression profile and thus do not correspond to the PBLs reaction.

The potential of phospho-H2AX foci scoring and the Comet assay as biomarkers for individual radiation sensitivity are controversially discussed. The assays were successfully used to identify DSB repair defective patients and patients with increased risk for high grade toxicities during radiotherapy [28], [29], [30], [31], [32]. Further reports showed correlations between the radiosensitive phenotype of knockout mice [33], several cancer cell lines [34], [35], [36], [37] or PBLs of one CT (computed tomography) -examined cancer patient [38] with residual γH2AX foci 24 h post irradiation. Also the Comet assay visualizes differences in the repair capacity of cell lines differing in their radiation sensitivities [39], [40], as well as between cancer patients and controls [41], [42], [43]. However, there are also studies challenging the predictive potentials of the yH2AX foci assay [44] and the Comet assay [45], [46]. In line with numerous other studies in which the radiation-induced histone H2AX phosphorylation was analysed in human PBLs [47], [48], [49], both laboratories showed a maximum of γH2AX-fluorescence after 1 h to 4 h and an almost complete repair 24 h later for both, PBLs and LCLs. As compared to PBLs, LCLs showed a higher basic H2AX-phosphorylation (data not shown) and thus confirmed the Comet assay data where the LCLs revealed significantly higher spontaneous and IR-induced DNA damage but a better repair capacity. The high basic γH2AX level of the LCLs is likely to be due to the proliferation of these cell types, since S-, G2- and M-phase cells have more DNA and thereby more histone protein to be phosphorylated. Furthermore, the synthesis of DNA during S-phase can lead to replication damage which could be marked by γH2AX, and a recent publication by McManus and Henzel [50] suggests a contribution of γH2AX to the fidelity of the mitotic process. However, Zijno et al. found LCLs to be not suitable for the analysis of γH2AX, especially when small inter-individual differences must be detected [51].

Although each participating laboratory was able to identify conspicuous individuals by both, γH2AX or Comet assay, the inter-laboratory comparison displayed no correlation, no matter whether PBLs or LCLs were used. A main difficulty lies in the large inter-individual variability in H2AX phosphorylation at early time points, which potentially conceals small changes resulting from differences in radiation sensitivity.

Therefore, it is highly questionable if the phosphorylation of H2AX is suitable to detect the slight differences in radiosensitivity among individual cancer patients. It might be possible that individuals with a pronounced defect in the radiation-response cascade may be retrieved as has been reported for knockout mice and well characterised cancer cell lines. However, the slight differences in the radiation-response of cancer patients might be undetectable with the γH2AX or Comet assay, at least under the described conditions. A retrospective power analysis revealed that with the present sample size we would be able to detect differences between clinical radiosensitive and non-radiosensitive patients if the effect size is greater than 1. Thus from our results it can be concluded that any effects – if they exist at all – are likely to have an effect size smaller than 1. Furthermore, specifically predefined endpoints are necessary to increase the probability of revealing effects. Table 6 shows the number of patients that is required to detect effects of different effect sizes. However, it should be kept in mind, that the 15 pairs of matched radiosensitive and non-radiosensitive samples analysed have been derived from more than 550 patients being treated and followed-up at the Department of Radiotherapy. To increase the sample size it is recommended to pool samples from different national and/or international studies.

Data of the investigated endpoints were not convincing to unequivocally predict radiation response. Thus, the predictive capacities of the analysed parameters have to be critically evaluated and future studies demand the investigation of new endpoints. Statistical analysis showed that PBLs reveal more reliable results than LCLs independently which biological endpoint was used. Concerning the Comet assay, OTM-data were more convincing than % Tail DNA. The evaluation of the data on a tumour-specific basis revealed that the values of all biological endpoints, except of the standardised % Tail DNA values, were significantly different in breast cancer samples compared to head and neck cancer patients. The reason for this interesting new finding is unclear and was not further investigated in this study, but it underlines the necessity to focus on one tumour entity in future studies.

Gene expression data of the PBLs turned out to be a promising predictive parameter. About 23,000 genes were analysed and a set of 67 genes was identified to be differentially up- or down-regulated after *in vitro* irradiation with 5 Gy and allowed to distinguish between the group of severely radiosensitive and non-radiosensitive patients (tables S1, S2, S3, S4, figure S1, array data are available under http://www.ncbi.nlm.nih.gov/geo/query/acc.cgi?acc=GSE40640 and a detailed description of the methods can be found in Methods to gene expression data S1). Detailed analysis showed 21 genes which were downregulated in radiosensitive but unchanged in normally reacting patients (group 2, table S1), 16 genes unchanged in sensitive but down regulated in normal cases (group 3, table S2), 19 genes unchanged in sensitive but up regulated in normal cases (group 4, table S3), and 11 genes upregulated in sensitive but unchanged in normal cases (group 5, table S4). Thus, our data reflect the complexity of the deviations in radiation response in patients with strong radiosensitivity. A considerable number of these genes belong to the apoptosis or cell cycle regulation pathway which have already been described to be involved in the radiation response [23]. As the sample number in our study is low, we applied restrictive selection criteria, e.g. the low error rate level of 2.5%, to control for false positive results. Thus, we rather would have missed genes than selected false positives. Moreover, because of the low sample number, we did not further scrutinize our signature for sensitivity and specificity although recommended by others [52]. We are however aware that our signature requires a comprehensive validation in larger sample sets. Although genome-wide expression changes in the radiation response were frequently studied in cell lines and tumour samples, studies comparing radiosensitive and normally reacting patients are rare. Thus, cohorts with careful clinical documentation of side effects for validation are limited or have to be established.

Nevertheless, three recent studies using gene expression profiles to predict radiotoxicity support our results. One study used PBLs of five head and neck cancer patients which were examined two weeks after chemo-radiation [53]. This study showed gene expression changes in PBLs during therapy and these changes were related to the degree of toxicity. The genes were involved in similar canonical pathways as those identified in our study. The largest study available compared radiation-induced gene expression in blood samples from 21 prostate cancer patients with severe side effects after radiotherapy with those from 17 normally reacting patients [54]. After irradiation of PBLs with 2 Gy, a classifying gene set was identified that could predict radiosensitivity in 63% of the patients. Again, the majority of classifying genes belonged to the apoptosis and stress signaling networks. Furthermore, constitutive gene expression levels of 81 genes were shown to predict toxicity in 12 breast cancer patients treated with high-dose hyper-fractionated radical radiotherapy [55]. The different results of the various studies presented are explained by the large divergence of radiation doses, kinetics, data evaluation, validation procedures, cancer types and clinical endpoints. Therefore, in future investigations this promising result of gene expression analysis as a reliable predictive parameter should be verified and confirmed on a larger patient collective and should be extended by further genetic and epigenetic analyses such as genotyping and determination of DNA methylation and microRNA regulation.

In conclusion, despite all efforts for standardisation, high inter-laboratory variabilities were observed. Because of the huge metabolic changes in response to the viral transfection, LCLs are not usable as surrogate for the radiobiological endpoints described, especially for apoptosis and DNA repair processes. Neither cell death nor radiation-induced DNA damage and repair were suitable parameters to predict normal tissue reaction. Also, the analysis based on the combination of different test results (e.g. apoptosis induction combined with DSB induction and repair) failed to distinguish between clinically radiosensitive and normal responding tumour patients. However, the outcome of our assays for apoptotic cell death, yH2AX foci and the Comet assay data revealed significant differences between the samples derived from breast cancer patients and the samples from head and neck cancer patients. Furthermore, in the hypersensitive cohort a set of 67 genes of the apoptosis and cell cycle regulation pathways showed a modified expression compared to the non-radiosensitive cohort and thus, expression analysis of these genes is an interesting tool to identify hypersensitive patients.

The advices for future studies are: 1) to use PBLs instead of LCLs, 2) to use higher radiation doses which might increase the differences between the individuals in the functional assays, 3) to investigate only one tumour entity, 4) to focus on gene expression, and 5) to critically assess single centre studies.

## Supporting Information

Figure S1 **Analysis of expression profiles in primary lymphocytes.** Primary lymphocytes derived from radiosensitive (yellow) and non-radiosensitive patients (green) were irradiated with 0 and 5 Gy and total RNA was collected after 6 h. The heat map represents the log2 fold changes of gene expression values (irradiated/untreated control). All 153 genes with radiation-induced fold changes >50% and adjusted p-values <0.025 in at least one of both patient groups are included. Red marks indicate radiation-induced upregulation, blue marks downregulation of gene expression; the colour intensity is a measure of the strength of regulation. Eighty-seven genes were down- or up- regulated in both patient groups (sensitivity differentiating gene groups 1 and 6, light grey and dark grey) and were therefore considered to be not informative. In contrast, 67 genes were differentially regulated after irradiation in the two patient groups and were thus suggested to classify radiosensitive from non-radiosensitive patients. Expression profiles are differing as follows: group 2 including 21 genes down-regulated (light grey) in radiosensitive but unchanged (grey) in normally reacting patients, group 3 including 16 genes unchanged (grey) in sensitive but down-regulated (light grey) in normal cases, group 4 including 19 genes unchanged (grey) in sensitive but up-regulated (dark grey) in normal cases, group 5 comprising 11 genes up-regulated (dark grey) in sensitive but unchanged (grey) in normal cases. Gene names are given in tables S1, S2, S3, S4 and in [23].
(TIF)

Table S1 Radiation-induced mRNA expression changes in genes differentially regulated in radiosensitive versus normally reacting patients: 21 genes down-regulated by irradiation in radiosensitive but not in normally reacting patients. Blood samples from 12 radiosensitive and 12 matched normally reacting patients were analysed. Selection criteria were a radiation-induced fold change >50% and an adjusted P value <0.025 in at least one group.
(DOC)

Table S2 Radiation-induced mRNA expression changes in genes differentially regulated in radiosensitive versus normally reacting patients: 16 genes down-regulated by irradiation in normally reacting but not in radiosensitive patients. Blood samples from 12 radiosensitive and 12 matched normally reacting patients were analysed. Selection criteria were a radiation-induced fold change >50% and an adjusted P value <0.025 in at least one group.
(DOC)

Table S3 Radiation-induced mRNA expression changes in genes differentially regulated in radiosensitive versus normally reacting patients: 19 genes up-regulated by irradiation in normally reacting but not in radiosensitive patients. Blood samples from 12 radiosensitive and 12 matched normally reacting patients were analysed. Selection criteria were a radiation-induced fold change >50% and an adjusted P value <0.025 in at least one group.
(DOC)

Table S4 Radiation-induced mRNA expression changes in genes differentially regulated in radiosensitive versus normally reacting patients: 11 genes up-regulated by irradiation in radiosensitive but not in normally reacting patients. Blood samples from 12 radiosensitive and 12 matched normally reacting patients were analysed. Selection criteria were a radiation-induced fold change >50% and an adjusted P value <0.025 in at least one group.
(DOC)

Methods to gene expression data S1 **The methods used for gene expression analysis.**
(DOC)
