# Supplementary figures and images for: Evaluation of Different Biomarkers to Predict Individual Radiosensitivity in an Inter-Laboratory Comparison–Lessons for Future Studies
Source: PLoS One. 2012 Oct 23;7(10):e47185. doi: 10.1371/journal.pone.0047185 (PMC3479094; doi:10.1371/journal.pone.0047185)

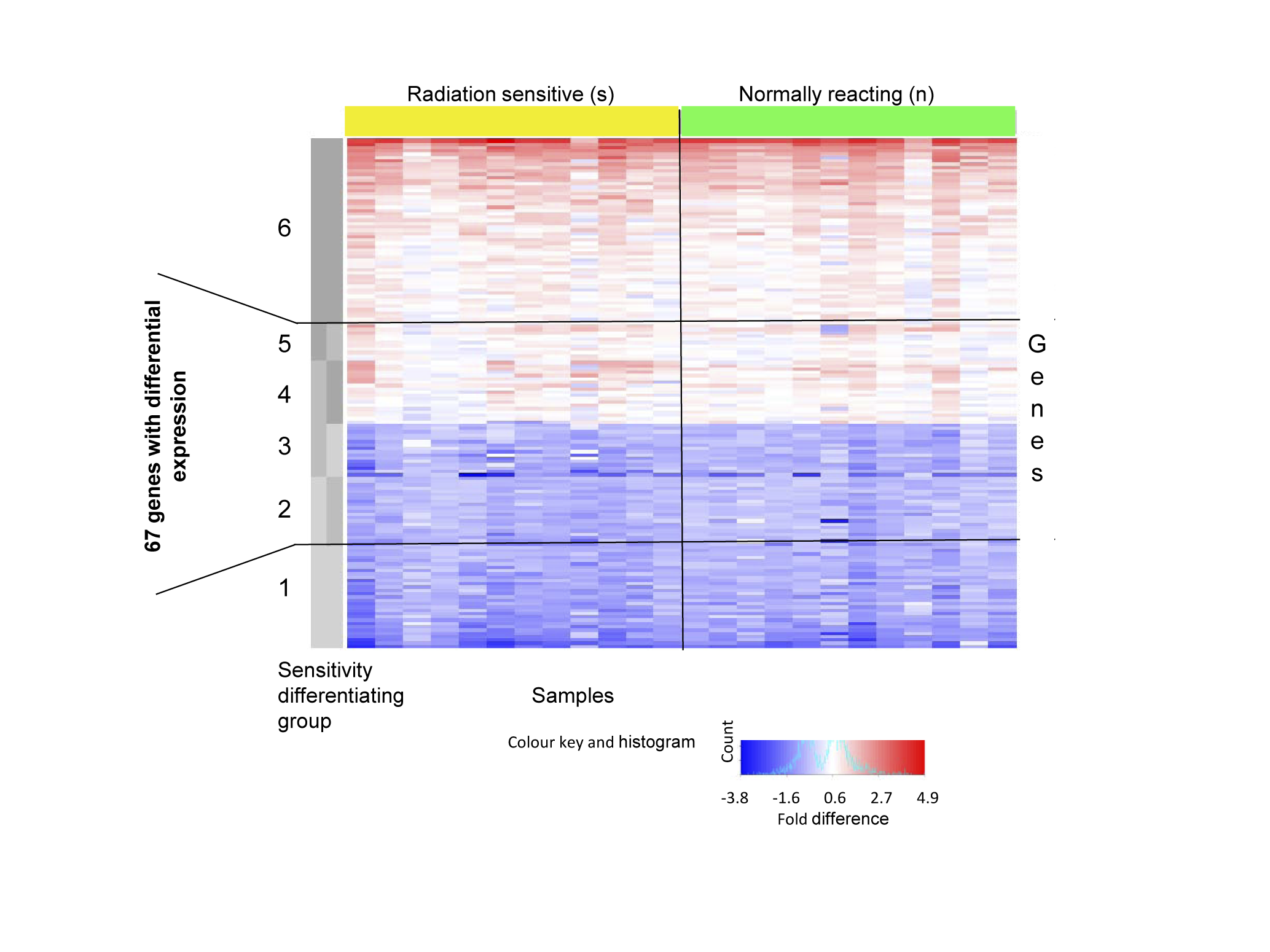

Supplement: Figure S1 — Analysis of expression profiles in primary lymphocytes. Primary lymphocytes derived from radiosensitive (yellow) and non-radiosensitive patients (green) were irradiated with 0 and 5 Gy and total RNA was collected after 6 h. The heat map represents the log2 fold changes of gene expression values (irradiated/untreated control). All 153 genes with radiation-induced fold changes >50% and adjusted p-values <0.025 in at least one of both patient groups are included. Red marks indicate radiation-induced upregulation, blue marks downregulation of gene expression; the colour intensity is a measure of the strength of regulation. Eighty-seven genes were down- or up- regulated in both patient groups (sensitivity differentiating gene groups 1 and 6, light grey and dark grey) and were therefore considered to be not informative. In contrast, 67 genes were differentially regulated after irradiation in the two patient groups and were thus suggested to classify radiosensitive from non-radiosensitive patients. Expression profiles are differing as follows: group 2 including 21 genes down-regulated (light grey) in radiosensitive but unchanged (grey) in normally reacting patients, group 3 including 16 genes unchanged (grey) in sensitive but down-regulated (light grey) in normal cases, group 4 including 19 genes unchanged (grey) in sensitive but up-regulated (dark grey) in normal cases, group 5 comprising 11 genes up-regulated (dark grey) in sensitive but unchanged (grey) in normal cases. Gene names are given in tables S1, S2, S3, S4 and in [23]. (TIF) [file pone.0047185.s001.tif]
