# Supplementary material for: Evaluation of Different Biomarkers to Predict Individual Radiosensitivity in an Inter-Laboratory Comparison–Lessons for Future Studies
Source: PLoS One. 2012 Oct 23;7(10):e47185. doi: 10.1371/journal.pone.0047185 (PMC3479094; doi:10.1371/journal.pone.0047185)
Supplement: Table S1 — Radiation-induced mRNA expression changes in genes differentially regulated in radiosensitive versus normally reacting patients: 21 genes down-regulated by irradiation in radiosensitive but not in normally reacting patients. Blood samples from 12 radiosensitive and 12 matched normally reacting patients were analysed. Selection criteria were a radiation-induced fold change >50% and an adjusted P value <0.025 in at least one group. (DOC) [file pone.0047185.s002.doc]

**Table S1**

| **Gene** | **Gene name** | **Chromosomal** | **Radiosensitive patients** | |  | **Normally reacting patients** | | |
| --- | --- | --- | --- | --- | --- | --- | --- | --- |
| **symbol** |  | **location** | **Fold change** | **Adjusted**  **P value** | **Score*** | **Fold change** | **adjusted P value** | **Score*** |
| HLA-DMB | major histocompatibility complex, class II, DM beta | 6p21.3 | 0.48 | 3.91E-03 | -1 | 0.56 | 2.57E-02 | 0 |
| BTK | Bruton agammaglobulinemia tyrosine kinase | Xq21.33-q22 | 0.52 | 6.47E-03 | -1 | 0.54 | 3.64E-01 | 0 |
| OSBPL10 | oxysterol binding protein-like 10 | 3p22.3 | 0.56 | 1.61E-05 | -1 | 0.68 | 1.93E-03 | 0 |
| GGA2 | golgi-associated, gamma adaptin ear containing, ARF binding protein 2 | 16p12 | 0.59 | 2.24E-02 | -1 | 0.66 | 2.53E-02 | 0 |
| IL16 | interleukin 16 | 15q26.3 | 0.60 | 1.07E-04 | -1 | 0.68 | 1.23E-03 | 0 |
| BIN1 | bridging integrator 1 | 2q14 | 0.62 | 6.33E-04 | -1 | 0.74 | 3.28E-02 | 0 |
| ITGB7 | integrin, beta 7 | 12q13.13 | 0.63 | 1.14E-02 | -1 | 0.75 | 7.93E-02 | 0 |
| CORO1A | coronin, actin binding protein, 1A | 16p11.2 | 0.63 | 1.10E-03 | -1 | 0.59 | 8.36E-01 | 0 |
| SPON1 | spondin 1, extracellular matrix protein | 11p15.2 | 0.63 | 8.54E-03 | -1 | 0.77 | 4.03E-03 | 0 |
| TMEM204 | transmembrane protein 204 | 16p13.3 | 0.63 | 1.68E-02 | -1 | 0.68 | 3.19E-02 | 0 |
| ARHGEF6 | Rac/Cdc42 guanine nucleotide exchange factor (GEF) 6 | Xq26.3 | 0.63 | 7.24E-04 | -1 | 0.70 | 5.14E-04 | 0 |
| ESYT1 | extended synaptotagmin-like protein 1 | 12q13.2 | 0.64 | 1.55E-05 | -1 | 0.67 | 7.01E-03 | 0 |
| SUN2 | Sad1 and UNC84 domain containing 2 | 22q13.1 | 0.64 | 3.26E-03 | -1 | 0.72 | 7.79E-02 | 0 |
| SH2D3C | SH2 domain containing 3C | 9q34.11 | 0.64 | 6.89E-03 | -1 | 0.69 | 2.09E-02 | 0 |
| CCND3 | cyclin D3 | 6p21 | 0.64 | 1.79E-03 | -1 | 0.68 | 4.95E-04 | 0 |
| INPP5D | inositol polyphosphate-5-phosphatase, 145kDa | 2q37.1 | 0.65 | 4.57E-05 | -1 | 0.69 | 1.23E-02 | 0 |
| PRKCB1 | protein kinase C, beta | 16p11.2 | 0.65 | 1.63E-02 | -1 | 0.71 | 5.14E-04 | 0 |
| SSH2 | slingshot homolog 2 (Drosophila) | 17q11.2 | 0.65 | 3.99E-03 | -1 | 0.75 | 2.80E-02 | 0 |
| FLOT2 | flotillin 2 | 17q11-q12 | 0.65 | 5.93E-04 | -1 | 0.71 | 1.15E-02 | 0 |
| AP3M2 | adaptor-related protein complex 3, mu 2 subunit | 8p11.2 | 0.66 | 4.07E-04 | -1 | 0.71 | 1.97E-03 | 0 |
| E2F5 | E2F transcription factor 5, p130-binding | 8q21.2 | 0.67 | 5.93E-04 | -1 | 0.73 | 9.40E-05 | 0 |

* Score: negative values indicate downregulation by irradiation, positive values upregultation, 0 represents no change in the respective patient group.
