# Supplementary material for: Evaluation of Different Biomarkers to Predict Individual Radiosensitivity in an Inter-Laboratory Comparison–Lessons for Future Studies
Source: PLoS One. 2012 Oct 23;7(10):e47185. doi: 10.1371/journal.pone.0047185 (PMC3479094; doi:10.1371/journal.pone.0047185)
Supplement: Table S2 — Radiation-induced mRNA expression changes in genes differentially regulated in radiosensitive versus normally reacting patients: 16 genes down-regulated by irradiation in normally reacting but not in radiosensitive patients. Blood samples from 12 radiosensitive and 12 matched normally reacting patients were analysed. Selection criteria were a radiation-induced fold change >50% and an adjusted P value <0.025 in at least one group. (DOC) [file pone.0047185.s003.doc]

**Table S2**

| **Gene** | **Gene symbol** | **Chromosomal** | **Radiosensitive patients** | | | **Normally reacting patients** | | |
| --- | --- | --- | --- | --- | --- | --- | --- | --- |
| **symbol** |  | **location** | **fold change** | **adjusted**  **P value** | **score*** | **fold change** | **adjusted P value** | **score*** |
| VPREB3 | pre-B lymphocyte 3 | 22q11 | 0.31 | 2.78E-02 | 0 | 0.35 | 9.25E-03 | -1 |
| HPCAL1 | hippocalcin-like 1 | 2p25.1 | 0.52 | 1.26E-01 | 0 | 0.64 | 1.70E-03 | -1 |
| RNASET2 | ribonuclease T2 | 6q27 | 0.54 | 5.90E-02 | 0 | 0.60 | 1.35E-02 | -1 |
| SIPA1 | signal-induced proliferation-associated 1 | 11q13 | 0.55 | 2.95E-02 | 0 | 0.57 | 9.57E-05 | -1 |
| IGHV5-78 | immunoglobulin heavy variable 5-78 (pseudogene) | 14q32.33 | 0.56 | 2.53E-02 | 0 | 0.62 | 2.50E-03 | -1 |
| LY9 | lymphocyte antigen 9 | 1q23.3 | 0.58 | 7.00E-02 | 0 | 0.61 | 6.61E-04 | -1 |
| FCRLA | Fc receptor-like A | 1q23.3 | 0.58 | 1.98E-01 | 0 | 0.67 | 3.19E-03 | -1 |
| ZNF395 | zinc finger protein 395 | 8p21.1 | 0.59 | 1.18E-01 | 0 | 0.66 | 1.85E-02 | -1 |
| PNPLA7 | patatin-like phospholipase domain containing 7 | 9q34.3 | 0.61 | 1.41E-01 | 0 | 0.63 | 3.91E-04 | -1 |
| LYL1 | lymphoblastic leukemia derived sequence 1 | 19p13.2 | 0.61 | 4.31E-02 | 0 | 0.63 | 9.84E-03 | -1 |
| PTPRCAP | protein tyrosine phosphatase. receptor type. C-associated protein | 11q13.3 | 0.61 | 6.99E-02 | 0 | 0.61 | 1.15E-02 | -1 |
| SUSD3 | sushi domain containing 3 | 9q22.31 | 0.68 | 1.47E-02 | 0 | 0.66 | 7.09E-05 | -1 |
| NMT2 | N-myristoyltransferase 2 | 10p13 | 0.68 | 2.83E-02 | 0 | 0.60 | 5.49E-03 | -1 |
| DEF6 | differentially expressed in FDCP 6 homolog (mouse) | 6p21.33-p21.1 | 0.69 | 4.33E-02 | 0 | 0.64 | 5.83E-03 | -1 |
| LAMA5 | laminin. alpha 5 | 20q13.2-q13.3 | 0.71 | 7.65E-02 | 0 | 0.66 | 3.53E-04 | -1 |
| SLC44A2 | solute carrier family 44. member 2 | 19p13.1 | 0.71 | 3.77E-02 | 0 | 0.62 | 4.78E-04 | -1 |

* Score: negative values indicate downregulation by irradiation. positive values upregultation. 0 represents no change in the respective patient group.
