# Supplementary material for: Evaluation of Different Biomarkers to Predict Individual Radiosensitivity in an Inter-Laboratory Comparison–Lessons for Future Studies
Source: PLoS One. 2012 Oct 23;7(10):e47185. doi: 10.1371/journal.pone.0047185 (PMC3479094; doi:10.1371/journal.pone.0047185)
Supplement: Table S3 — Radiation-induced mRNA expression changes in genes differentially regulated in radiosensitive versus normally reacting patients: 19 genes up-regulated by irradiation in normally reacting but not in radiosensitive patients. Blood samples from 12 radiosensitive and 12 matched normally reacting patients were analysed. Selection criteria were a radiation-induced fold change >50% and an adjusted P value <0.025 in at least one group. (DOC) [file pone.0047185.s004.doc]

**Table S3**

| **Gene** | **Gene name** | **Chromosomal** | **Radiosensitive patients** | | | **Normally reacting patients** | | |
| --- | --- | --- | --- | --- | --- | --- | --- | --- |
| **symbol** |  | **location** | **Fold change** | **Adjusted**  **P value** | **Score*** | **Fold change** | **adjusted P value** | **Score*** |
| IFNG | interferon, gamma | 12q14 | 1.171 | 1.00E+00 | 0 | 1.683 | 1.15E-02 | 1 |
| CDS2 | CDP-diacylglycerol synthase (phosphatidate cytidylyltransferase) 2 | 20p13 | 1.325 | 7.37E-02 | 0 | 1.502 | 1.15E-02 | 1 |
| PUS7 | pseudouridylate synthase 7 homolog (S. cerevisiae) | 7q22.3 | 1.334 | 1.84E-01 | 0 | 1.541 | 2.79E-03 | 1 |
| TRIM32 | tripartite motif containing 32 | 9q33.1 | 1.397 | 4.87E-03 | 0 | 1.511 | 1.88E-03 | 1 |
| HIST1H4H | histone cluster 1, H4h | 6p22.1 | 1.452 | 1.32E-01 | 0 | 1.502 | 1.01E-02 | 1 |
| RPL34 | ribosomal protein L34 | 4q25 | 1.475 | 4.87E-02 | 0 | 1.512 | 8.69E-03 | 1 |
| IKIP | IKBKB interacting protein | 12q23.1 | 1.491 | 8.46E-02 | 0 | 1.544 | 8.47E-04 | 1 |
| GRHPR | glyoxylate reductase/hydroxypyruvate reductase | 9q12 | 1.497 | 3.63E-03 | 0 | 1.502 | 1.95E-04 | 1 |
| FAM186B | family with sequence similarity 186, member B | 12q13.12 | 1.499 | 7.00E-02 | 0 | 1.563 | 2.31E-03 | 1 |
| FCER2 | Fc fragment of IgE, low affinity II, receptor for (CD23) | 19p13.3 | 1.551 | 1.12E-01 | 0 | 1.530 | 1.85E-02 | 1 |
| SLC7A1 | solute carrier family 7 (cationic amino acid transporter, y+ system), member 1 | 13q12-q14 | 1.704 | 2.83E-02 | 0 | 1.512 | 5.74E-03 | 1 |
| APOBEC3H | apolipoprotein B mRNA editing enzyme, catalytic polypeptide-like 3H | 22q13.1 | 1.741 | 3.79E-02 | 0 | 1.827 | 5.73E-04 | 1 |
| NFKB2 | nuclear factor of kappa light polypeptide gene enhancer in B-cells 2 (p49/p100) | 10q24 | 1.743 | 1.27E-01 | 0 | 1.591 | 1.06E-03 | 1 |
| BCL2L1 | BCL2-like 1 | 20q11.21 | 1.768 | 2.81E-02 | 0 | 1.743 | 1.46E-03 | 1 |
| PSAT1 | phosphoserine aminotransferase 1 | 9q21.2 | 1.847 | 8.38E-02 | 0 | 1.761 | 2.23E-03 | 1 |
| ABTB2 | ankyrin repeat and BTB (POZ) domain containing 2 | 11p13 | 1.893 | 2.98E-02 | 0 | 1.808 | 1.86E-02 | 1 |
| NME1 | non-metastatic cells 1, protein (NM23A) expressed in | 17q21.3 | 1.952 | 3.29E-02 | 0 | 2.204 | 3.57E-04 | 1 |
| CD80 | **CD80** molecule | 3q13.3-q21 | 2.153 | 4.01E-02 | 0 | 1.880 | 9.00E-03 | 1 |
| IRF4 | interferon regulatory factor 4 | 6p25-p23 | 2.178 | 3.75E-02 | 0 | 1.641 | 3.20E-03 | 1 |

* Score: negativ values indicate downregulation by irradiation. positive values upregultation. 0 represents no change in the respective patient group.
