# Supplementary material for: Evaluation of Different Biomarkers to Predict Individual Radiosensitivity in an Inter-Laboratory Comparison–Lessons for Future Studies
Source: PLoS One. 2012 Oct 23;7(10):e47185. doi: 10.1371/journal.pone.0047185 (PMC3479094; doi:10.1371/journal.pone.0047185)
Supplement: Table S4 — Radiation-induced mRNA expression changes in genes differentially regulated in radiosensitive versus normally reacting patients: 11 genes up-regulated by irradiation in radiosensitive but not in normally reacting patients. Blood samples from 12 radiosensitive and 12 matched normally reacting patients were analysed. Selection criteria were a radiation-induced fold change >50% and an adjusted P value <0.025 in at least one group. (DOC) [file pone.0047185.s005.doc]

**Table S4**

| **Gene** | **Gene name** | **Chromosomal** | **Radiosensitive patients** | | | **Normally reacting patients** | | |
| --- | --- | --- | --- | --- | --- | --- | --- | --- |
| **symbol** |  | **location** | **Fold change** | **Adjusted**  **P value** | **Score*** | **Fold change** | **adjusted P value** | **Score*** |
| CYB5A | cytochrome b5 type A (microsomal) | 18q23 | 1.51 | 6.20E-03 | 1 | 1.39 | 1.15E-02 | 0 |
| ISCU | iron-sulfur cluster scaffold homolog (E. coli) | 12q24.1 | 1.52 | 1.18E-02 | 1 | 1.46 | 1.26E-03 | 0 |
| POPDC2 | popeye domain containing 2 | 3q13.33 | 1.52 | 7.04E-03 | 1 | 1.46 | 1.09E-03 | 0 |
| PLEKHG1 | pleckstrin homology domain containing, family G (with RhoGef domain) member 1 | 6q25.1 | 1.52 | 3.08E-03 | 1 | 1.45 | 4.10E-03 | 0 |
| EDA2R | ectodysplasin A2 receptor | Xq12 | 1.52 | 2.83E-03 | 1 | 1.43 | 7.64E-05 | 0 |
| TMEM88 | transmembrane protein 88 | 17p13.1 | 1.56 | 4.61E-03 | 1 | 1.37 | 1.52E-02 | 0 |
| RCN1 | reticulocalbin 1, EF-hand calcium binding domain | 11p13 | 1.56 | 8.75E-03 | 1 | 1.45 | 1.85E-02 | 0 |
| PLK3 | polo-like kinase 3 | 1p34.1 | 1.58 | 2.74E-03 | 1 | 1.44 | 6.17E-02 | 0 |
| BAX | BCL2-associated X protein | 19q13.3-q13.4 | 1.85 | 5.89E-03 | 1 | 1.72 | 3.12E-02 | 0 |
| CD40 | CD40 molecule, TNF receptor superfamily member 5 | 20q12-q13.2 | 1.98 | 3.08E-03 | 1 | 1.92 | 1.08E-01 | 0 |
| TRAF4 | TNF receptor-associated factor 4 | 17q11-q12 | 2.02 | 1.73E-02 | 1 | 1.76 | 1.25E-01 | 0 |

* Score: negativ values indicate downregulation by irradiation. positive values upregultation. 0 represents no change in the respective patient group.
