# Supplementary material for: Evaluation of Different Biomarkers to Predict Individual Radiosensitivity in an Inter-Laboratory Comparison–Lessons for Future Studies
Source: PLoS One. 2012 Oct 23;7(10):e47185. doi: 10.1371/journal.pone.0047185 (PMC3479094; doi:10.1371/journal.pone.0047185)
Supplement: Methods to gene expression data S1 — The methods used for gene expression analysis. (DOC) [file pone.0047185.s006.doc]

*Method for whole genome expression analysis (Microarray)*

Total RNA was isolated from PBLs and EBV-transformed cell lines before and 6 h after irradiation with 5 Gy using Trizol (Invitrogen, Karlsruhe, Germany). Preparation of biotin labeled cRNA samples, hybridization of samples to arrays, and development of hybridization signals was performed according to Illumina´s standard procedures (Illumina, Inc.; San Diega, CA, USA). For hybridization, Human Sentrix-8 Beadchips arrays comprising 22,177 genes (Illumina) were used. Microarray scanning was done using a Beadstation array scanner, setting adjusted to a scaling factor of 1 and PMT settings at 430. Raw data were extracted using Illumina's BeadStudio software. After exclusion of outliers and removal of background, an average intensity value was calculated for each bead type. Data were corrected for differences in expression levels across a chip and between chips by applying quantile normalisation of log2 transformed data. This procedure yielded gene expression values used for further analyses [23]. Array data are available under <http://www.ncbi.nlm.nih.gov/geo/query/acc.cgi?acc=GSE40640>.

Evaluable complete data sets for mRNA expression with and without irradiation in primary and immortalised cells were obtained for 12 pairs of patients. To analyze gene expression data with regard to the patient's clinical reaction to the radiotherapy, clinical patient data were decoded. In order to check the radiation effect in cells of radiosensitive and normal reacting patients, two-factorial linear regression models were applied for primary and immortalized cells. *P* values were calculated for empirical Bayes moderated *t*-statistics . Resulting *p* values were adjusted according to control the false discovery rate. As statistical analyses were done separately for the two patient groups, sensitive or normal reacting, a global significance level of α=2.5% was selected. All statistical calculations for array evaluation were performed using R version 2.7.2, and R/Bioconductor packages beadarray, multtest and limma. The DAVID Bioinformatics Resources tool, version 6.7, was applied to perform a gene-enrichment and functional annotation analysis for the 67 genes differentiating radiosensitive and normal reacting patients.
